# TEM-based Study of the Phenotype of Astrocytes Differentiated from Induced Pluripotent Stem Cells from a Healthy Donor and a Patient with Parkinson’s Disease

**DOI:** 10.17691/stm2026.18.1.01

**Published:** 2026-02-27

**Authors:** K.A. Kutukova, M.V. Ivanov, E.V. Novosadova, A.V. Brydun, E.L. Arsenyeva, L.V. Novosadova, I.V. Kokorev, I.A. Grivennikov, V.S. Sukhorukov, S.N. Illarioshkin

**Affiliations:** Junior Researcher, Laboratory of Neuromorphology, Institute of Brain Research; Russian Research Center of Neurology and Neurosciences, 80 Volokolamskoye Shosse, Moscow, 125367, Russia; Senior Lecturer, Department of Morphology, Institute of Anatomy and Morphology; Pirogov Russian National Research Medical University, 1 Bldg., 6 Ostrovityanova St., Moscow, 117513, Russia; Junior Researcher, Laboratory of Neuromorphology, Institute of Brain Research; Russian Research Center of Neurology and Neurosciences, 80 Volokolamskoye Shosse, Moscow, 125367, Russia; Senior Lecturer, Department of Morphology, Institute of Anatomy and Morphology; Pirogov Russian National Research Medical University, 1 Bldg., 6 Ostrovityanova St., Moscow, 117513, Russia; PhD, Senior Researcher, Laboratory of Cell Differentiation; National Research Center “Kurchatov Institute”, 1 Academician Kurchatov Square, Moscow, 123182, Russia; Leading Researcher, Laboratory of Translational Biomedicine; Lopukhin Federal Research and Clinical Center of Physical-Chemical Medicine of Federal Medical Biological Agency, 1a Malaya Pirogovskaya St., Moscow, 119435, Russia; MD, PhD, Researcher; Russian Research Center of Neurology and Neurosciences, 80 Volokolamskoye Shosse, Moscow, 125367, Russia; Head of the Educational Laboratory, Department of Morphology, Institute of Anatomy and Morphology; Pirogov Russian National Research Medical University, 1 Bldg., 6 Ostrovityanova St., Moscow, 117513, Russia; PhD, Chief Specialist; National Research Center “Kurchatov Institute”, 1 Academician Kurchatov Square, Moscow, 123182, Russia; Engineer, Grade 1; National Research Center “Kurchatov Institute”, 1 Academician Kurchatov Square, Moscow, 123182, Russia; Student, Department of Biotechnology and Industrial Pharmacy; MIREA — Russian Technological University, 78 Vernadsky Avenue, Moscow, 119454, Russia; DSc, Professor, Chief Researcher, Laboratory of Molecular Neurogenetics and Natural Immunity; National Research Center “Kurchatov Institute”, 1 Academician Kurchatov Square, Moscow, 123182, Russia; MD, DSc, Professor, Head of Laboratory of Neuromorphology, Institute of Brain Research; Russian Research Center of Neurology and Neurosciences, 80 Volokolamskoye Shosse, Moscow, 125367, Russia; Professor, Department of Morphology, Institute of Anatomy and Morphology; Pirogov Russian National Research Medical University, 1 Bldg., 6 Ostrovityanova St., Moscow, 117513, Russia; MD, DSc, Professor, Academician of the Russian Academy of Sciences, Director of Institute of Brain Research; Russian Research Center of Neurology and Neurosciences, 80 Volokolamskoye Shosse, Moscow, 125367, Russia

**Keywords:** Parkinson’s disease, induced pluripotent stem cells, differentiation, electron microscopy, astrocytes, *LRRK2*

## Abstract

**The aim of this study** was to study the role of transmission electron microscopy (TEM) in assessment of the phenotype of astrocytes obtained with the directed differentiation technique from induced pluripotent stem cells (iPSCs) from a healthy donor and from a patient with a hereditary form of Parkinson’s disease (PD).

**Materials and Methods:**

Monolayer astrocyte cultures differentiated from iPSCs from a healthy donor and a PD patient having the G2019S mutation in the *LRRK2* gene were used in the study. The obtained glial cultures were characterized using real-time PCR and immunocytochemical staining for glia-specific genes and proteins. TEM was used to examine astrocyte ultrastructure.

**Results:**

PCR analysis and immunocytochemical staining demonstrated that cell lines received from a healthy donor and a PD patient expressed the required pattern of glia-specific genes and synthesized astrocyte-specific proteins. However, some glia-specific genes were expressed at reduced levels by mutant cells. One of the most typical ultrastructural features of astrocytes received from iPSCs from a PD patient was destructive changes in mitochondria, including mitochondrial clearing, swelling, and cristae destruction. In many cells, mitochondria were completely absent after a long culturing. Another characteristic feature of cells with a mutation in the *LRRK2* gene was the accumulation of vacuoles with contents of varied electron density. Distinct changes in the ultrastructure of nuclei, protein-synthesizing organelles, and cytoskeletal elements were also seen in cultured astrocytes with a PD-associated *LRRK2* mutation. Here, the morphometric study did not reveal any differences in the average cell area, nuclear area, cytoplasm area, or nuclear-cytoplasmic ratio between astrocytes of the control line and the PD mutation line.

**Conclusion:**

Reprogramming and obtaining of astrocytes from iPSCs received from a donor with a PD-associated mutation in the *LRRK2* gene allow to assess the nature and dynamics of pathological morphochemical and ultrastructural changes caused by the mutation during gliogenesis. The use of combined techniques (PCR, immunocytochemistry, TEM) to compare cell cultures differentiated from iPSCs allow to assess, on the one hand, general culture parameters, such as the dynamics of culture differentiation based on changes in the expression level of specific genes and immunocytochemical markers, and on the other hand, morphofunctional changes at the level of individual cells. TEM demonstrates significant potential for studying cell cultures differentiated from iPSCs. This technique is instrumental for phenotyping the resulting cells based on their ultrastructure, assessing the degree of their morphological maturity, and identifying minor ultrastructural changes in cells, both pathological and differentiation-associated. The results of this TEM-based study indicate a pronounced decrease in mitochondrial viability and other ultrastructural abnormalities, thus confirming the idea of a significant role of astroglia in the development of the neurodegenerative process in the *LRRK2*-associated PD; hence, astroglia can be a basis for development of new approaches as well as for searching pharmacological targets in the pathogenetic therapy of the disease.

## Introduction

Parkinson’s disease (PD) is the second most common neurodegenerative disorder (next to Alzheimer’s disease). It is characterized by progressive motor dysfunction (hypokinesia, muscle rigidity, resting tremor), which is morphologically expressed in degeneration of the substantia nigra of the midbrain, with predominant loss of dopaminergic neurons. Most cases of PD are sporadic, but approximately 10% of patients have monogenic forms of the disease, inherited in an autosomal dominant or autosomal recessive manner [1]. The most significant genes associated with the development of PD include the *LRRK2*, *SNCA*, *PARK2*, *DJ-1*, *PINK1*, *GBA*, *VPS35*, and *ATP13A2* genes [2]. For instance, mutations in the *LRRK2* gene, which encodes the dardarin protein, cause one of the familial forms of PD with late onset, the clinical and pathological manifestations of which are similar to those of the more common sporadic form of PD [3, 4]. In case of pathogenic G2019S mutation in the *LRRK2* gene, the kinase activity of dardarin increases [5]. Morphological manifestations of mutations in the *LRRK2* gene in neurons include shortening of processes, simplification of the dendritic tree [6], as well as intracellular changes related to impaired macroautophagy [7–11].

Currently, the PD-associated disorders of neuroglia and glia interactions get increasingly greater attention. For instance, it was found that mutations in the *LRRK2* gene are associated not only with impaired intracellular utilization of the alpha-synuclein protein in neurons, but also with a decreased ability of astrocytes to internalize extracellular alpha-synuclein through endocytosis, which enters the extracellular space as a result of neuronal death [12]. Moreover, it is known that activated astrocytes in the brain are involved in the initiation of neuroinflammation associated with neurodegeneration [13]. The technique that allows to obtain glial cells from iPSCs boosted the possibilities for studying their role in neurogenesis and in the pathogenesis of neurodegenerative diseases, including PD [14, 15]. In [16], the immunofluorescence and the electron microscopy techniques were used to demonstrate the violation of exosome biogenesis in iPSC-derived astrocytes with the G2019S mutation in the *LRRK2* gene. di Domenico et al. [17] identified abnormalities in chaperone-mediated mitophagy, macroautophagy, and alpha-synuclein accumulation in astrocytes with this mutation.

Therefore, identification of the possible role of astrocytes in PD pathogenesis by means of a comprehensive study of their morphofunctional changes, including at the ultrastructural level, is currently of particular relevance.

**The aim of this study** was to compare astrocytes obtained from iPSCs from a healthy donor and a patient with a hereditary *LRRK2*-associated form of PD using a combination of techniques, including real-time polymerase chain reaction (PCR), immunocytochemical staining, and transmission electron microscopy (TEM).

## Materials and Methods

### Cell lines

The study was conducted with monolayer cultures of astrocytes differentiated from iPSCs from a healthy donor and a PD patient having the G2019S mutation in the *LRRK2* gene [18]. The study was approved by the Ethics Committee of the Russian Center for Neurology and Neuroscience (Russia) and was conducted in accordance with the ethical principles established by the Declaration of Helsinki (2024). Each cell donor submitted a written informed consent.

### Directed differentiation of neuronal progenitors into glial cells

The study protocol is based on the guidelines described in [19]. Neuronal progenitors derived from iPSCs were seeded onto 35 mm Petri dishes coated with Matrigel (BD Biosciences, USA) with 250,000–400,000 cells per 1 cm^2^ and cultured in a CO_2_ incubator at 37°C for 24 h in a medium for neuronal progenitors. The medium consisted of Neurobasal Medium (Gibco, USA), 2% SR (Gibco, USA), 1% N2 supplement (Gibco, USA), 1% B-27 supplement (PanEco, Russia), 2 mM glutamine (ICN Biomedicals, USA), 1% amino acid mixture (PanEco, Russia), 50 U/ml penicillin–streptomycin (PanEco, Russia), 80 ng/ml Noggin (PeproTech, USA), and 10 μM SB431542 (Stemgent, Inc., USA). The next day, the neuronal medium was replaced with the glial cell culture medium (DMEM/F12 (Gibco, USA), 1% NEAA (Hyclone, USA), glutamine 2 mm (PanEco, Russia), N2, B27, FGF2 8 ng/ml, Heregulin 10 ng/ml, IGF1200 ng/ml, Activin A 10 ng/ml (Stem Cell Technologies, Canada), penicillin–streptomycin 50 μg/ml (PanEco, Russia)). As the cells grew to a full monolayer, they were subcultured at a 1:3 ratio using 0.05% trypsin solution. The medium was changed every other day.

### Real-time polymerase chain reaction

PCR amplification was conducted using a 5X ready-made reaction mixture (Eurogen, Russia) containing the following components: Taq DNA polymerase, a mixture of nucleotide triphosphates, Mg^2+^, and a reaction buffer. An intercalating dye (SYBR Green) was used for real-time PCR. The reaction was conducted in a LightCycler® 96 amplifier (Roche, Germany) according to the manufacturer’s instructions. For PCR amplification of the reverse transcription reaction products, the authors used 0.04 portions of the reaction mixture (complementary DNA) — 1 μl; 5X qPCRmix-HS reaction mixture (Eurogen, Russia) — 5 μl; primers (see the Table) (Eurogen, Russia) — 1 μl each; deionized water (mQ) — to get a final volume of 25 μl.

**Table:** Primers used in the study

Gene	Primer nucleotide sequence	Touch down temperature (°C)
*GLUT1*	GCTGTGCTTATGGGCTTCTC CACATACATGGGCACAAAGC	60
*VIM*	ATTCCACTTTGCGTTCAAGG CTTCAGAGAGAGGAAGCCGA	60
*AQP4*	GGCCGTAATCTGACTCCCAG TGTGGGTCTGTCACTCATGC	60
*PLP1*	GGCGCAGTCAGGCAGATC CCCTTGCCGCAGATGGT	60
*MBP*	CTATAAATCGGCTCACAAGG AGGCGGTTATATTAAGAAGC	60
*CNP*	CACCATGCACCTCTCCCAGC ATGGAGCCGATCCGGTCCAG	60
*S100B*	CTGGAGAAGGCCATGGTTGC CTCCAGGAAGTGAGAGAGCT	60
*EAAT1*	CGAAGCCATCATGAGACTGGTA TCCCAGCAATCAGGAAGAGAA	60
*GAT3*	CTGATACGAGGGGTCACGTT GTTCAGGCAACAGAGCATGA	65
*ALDHIL1*	TCACAGAAGTCTAACCTGCC AGTGACGGGTGATAGATGAT	55.5
*SOX10*	AGTACCCGCACCTGCACA GAAGGGGCGCTTGTCACT	57
*GFAP*	TCCTGGAACAGCAAAACAAG CAGCCTCAGGTTGGTTTCAT	60
*BDNF*	ATTGGCTGGCGATTCATAAG GTTTCCCTTCTGGTCATGGA	60
*NT3*	AACTGCTGCGACAACAGAGA CCAGCCCACGAGTTTATTGT	60
*GDNF*	ACCTGGAGTTAATGTCCAACC GGCATATTTGAGTCACTGCT	60

### Fixing cells with paraformaldehyde and immunocytochemical analysis

Cells were washed once with PBS (ICN Biomedicals, USA) and fixed in 4% paraformaldehyde (Sigma, USA) for 20 min at room temperature. After washing three times with PBS, the cells were incubated in PBS added with 0.1% Triton X-100 and 5% fetal bovine serum (FBS) for 15 min to permeabilize them and decrease nonspecific antibody adsorption. Then, the cells were incubated in PBS added with 0.1% Triton X-100 and 5% FBS, which contained specific primary antibodies, at the concentrations recommended by the manufacturer, overnight at 4°C. After three 10-minute washes with PBS containing 0.1% Tween 20, the cells were incubated in PBS containing 0.1% Triton X-100 and 5% FBS with fluorescently labeled secondary antibodies at the manufacturer’s recommended concentrations for 1.5 h at room temperature. After three 10-minute washes with PBS containing 0.1% Tween 20, DAPI nuclear stain (Sigma, USA) was added to the cells at the concentration of 0.1 μg/ml, the cells were incubated for 20 min, and then washed three times with PBS containing 0.1% Tween 20. The preparations were visualized using an Imager Z1 fluorescence microscope (Zeiss, Germany). Images were analyzed using the ImageJ 1.49p software (NCBI, USA).

Primary antibodies used in the study:

Mouse Anti-VGLUT (MAB5502; Sigma-Aldrich, USA), 1:30;
Rabbit Anti-SLC1A3 (EAAT1) (MBS136184; My Bio Source, USA), 1:100;
Rabbit Anti-NG2 (ab 83178; Abcam, UK), 1:50; Rabbit Anti-CD44 (GTX102111; GeneTex, USA), 1:300;
Rabbit Anti-S100 (IR50461–2; Dako, Denmark), 1:2. Secondary antibodies used in the study:
Goat anti-mouse Alexa Fluor 488 (A11001; Invitrogen, USA), 1/2000;
Goat anti-rabbit Alexa Fluor 546 (A11010; Invitrogen, USA), 1/2000.

### Transmission electron microscopy

TEM examination was conducted with cell cultures obtained from a healthy donor and cultured for 25 days *in vitro* (DIV 25), as well as cells from a PD patient with a mutation in the *LRRK2* gene of the same culture period (DIV 26). In addition, the authors examined cells from a PD patient of the same lineage, but at a shorter differentiation period (DIV 10). Cells were fixed with 2.5% glutaraldehyde in 0.1 M phosphate buffer (pH 7.4) for 2 h, postfixed in 1% OsO_4_ solution for 2 h, dehydrated in ascending ethanol concentrations, and embedded in the EPON epoxy resin (Fluka, Switzerland). Ultrathin sections were obtained using an 8800 Ultratome III device (LKB Bromma, Sweden), contrasted with uranyl acetate and lead citrate, and examined using a JEM-1011 transmission electron microscope (JEOL, Japan) equipped with an ES500W Model 782 camera (Gatan Erlangshen, China). Morphometry was performed using the ImageJ software with digital images of sections of the same magnification. The areas of profile fields of cells, nuclei, and mitochondria were measured; mitochondrial circularity and the area of mitochondria were assessed relative to the cell cytoplasm. Mitochondria were included in the morphometric analysis if they contained at least one distinct crista.

### Statistical analysis of results

Data processing and statistical analysis were conducted using GraphPad Prism 8 and Statistica 8. The asymmetry and kurtosis tests were used to assess the distribution of data. Statistical analysis of the PCR data was performed using an unpaired, two-tailed t test. Differences were considered statistically significant at p<0.05. For the morphometric electron microscopic study, differences between the studied samples were identified using the nonparametric Mann–Whitney test. Differences were considered statistically significant at p<0.05.

## Results

### Polymerase chain reaction and immuno-cytochemistry

The resulting glial cultures were characterized using real-time PCR and immunocytochemistry staining.

**Figure 1.**
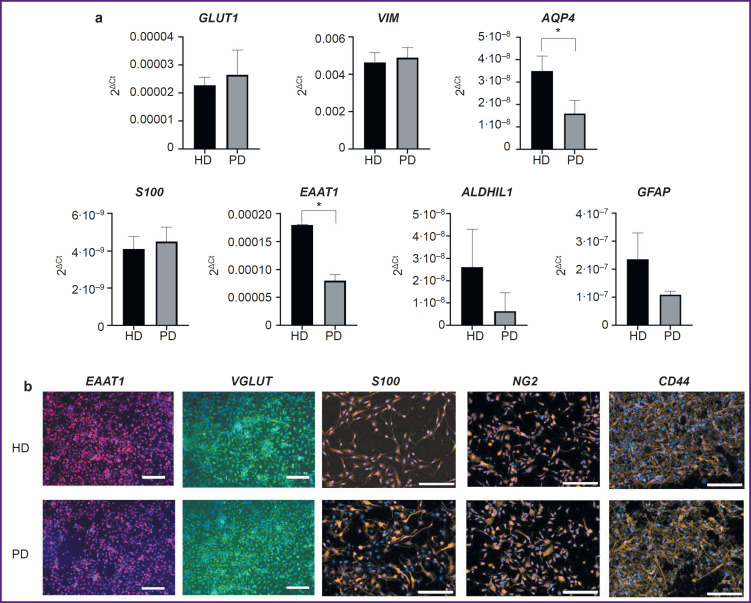
Results of polymerase chain reaction and immunocytochemical staining of astroglia cultures obtained from a healthy donor (HD) and from a patient with a mutation in the *LRRK2* gene (PD): (a) expression of glia-specific genes in cells; results are shown as the mean ± SEM; statistical analysis was conducted using an unpaired two-tailed t test; ^*^ statistically significant differences between groups, p<0.05; (b) immunocytochemical staining of glial cells with antibodies to *EAAT1*, *VGLUT*, *S100*, *NG2*, *CD44* and the nuclear dye DAPI; bar for *EAAT1* and *VGLUT* is 100 μm, for others — 200 μm

Both cell lines expressed the required pattern of glia-specific genes and synthesized glia-specific proteins (Figure 1). Here, some glia-specific genes (*AQP4*, *EAAT1*) were expressed at reduced levels in cultures with the *LRRK2* gene mutation compared to controls.

### Ultrastructure of astrocytes differentiated from iPSCs of a conditionally healthy donor

**Figure 2.**
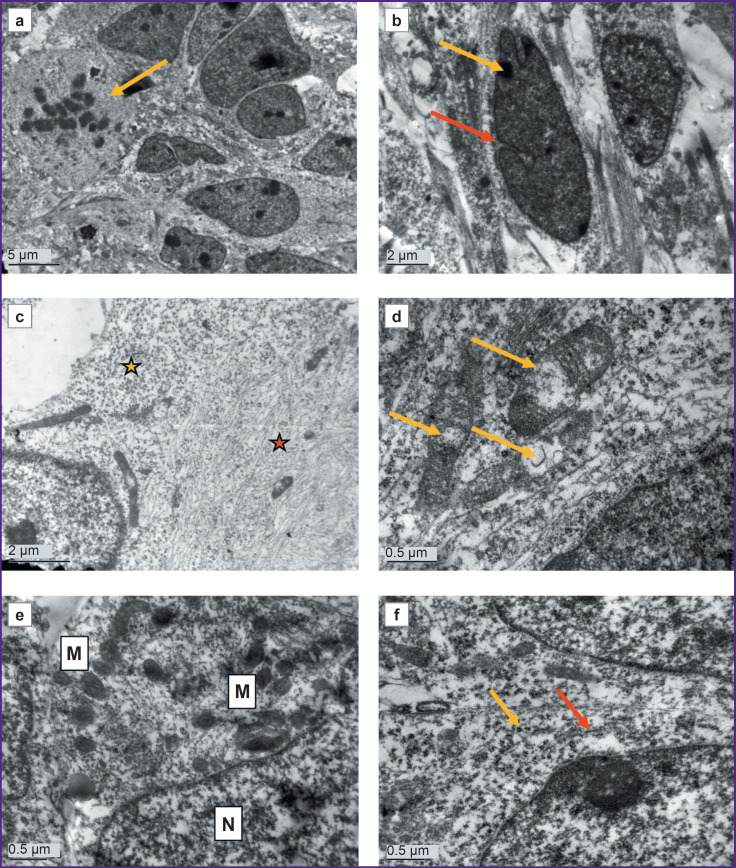
Electron microscopy of astrocyte culture (DIV 25), obtained from cell material from a healthy donor: (a) overview of the culture; dividing cells are seen (*yellow arrow*); (b) a typical cell nucleus with an eccentrically located nucleolus (*yellow arrow*) and shallow invaginations (*red arrow*); (c) different ratios of cytoskeletal elements and organelles in the cell: an area with a lower number of intermediate filaments and a higher number of polysomes and mitochondria (*yellow star*) and an area with a higher number of intermediate filaments and a lower number of organelles (*red star*); (d) mitochondria with regular cristae and areas of clearing and swelling (*yellow arrows*); (e) small round mitochondria aggregated in the perinuclear area of the cytoplasm; M — mitochondria, N — nucleus; (f) the protein-synthesizing apparatus is represented by polysomes (*yellow arrow*) and several short cisterns of the granular endoplasmic reticulum (*red arrow*)

Control astrocyte cultures had uniform cells with moderately electron-clear cytoplasm and clear nuclei. There were dividing cells, with virtually no dying cells (Figure 2 (a)). Cell nuclei were predominantly moderately electron-clear, with evenly distributed chromatin and a prominent, eccentrically located nucleolus. Small, evenly distributed clumps of heterochromatin were found in the nucleus. Shallow invaginations were seen in the nuclei of some cells (Figure 2 (b)). The cytoplasm was poorly populated with organelles and primarily filled with a fine network of intermediate filaments and polysomes (see Figure 2 (b); Figure 2 (c)). The hyaloplasm was characterized by electron transparency, which is also typical of normal human brain astrocytes. The cytoskeleton constituted the main element of the cell ultrastructure and consisted of a network of fine threads forming intermediate filaments. In some cells, the cytoskeleton was uniformly distributed, whereas in other cells, areas with denser and sparser clusters of filaments were found. A chaotic orientation of filaments was seen in the cell body, whereas in the processes, the filaments were arranged in parallel to each other. There were cells in which one part of the cytoplasm was filled with a dense network of filaments with a small number of polysomes in between, while another part, on the contrary, had predominating polysomes and contained few cytoskeletal elements (see Figure 2 (c)). Probably, the areas of the cytoplasm with a large number of polysomes were areas in which active production of proteins for cytoskeletal construction was only started. Mitochondria were predominantly elongated, with an electron-dense matrix and regular cristae. In specific mitochondria, loci of clearing and swelling were seen; they were located either in the center or at the end of the organelle (Figure 2 (d)). Modified mitochondria with destructed cristae were also observed. Mitochondria were few in number, scattered individually or aggregated near the nucleus or at the sites of processes branching (Figure 2 (e)). Isolated contacts of mitochondria with each other and with the nucleus were identified. The protein-synthesizing apparatus consisted predominantly of free ribosomes and polysomes. Distinct short cisternae of the granular endoplasmic reticulum (ER) were rare (Figure 2 (f)). The Golgi apparatus was poorly developed and had sporadic dictyosomes in it.

Cell processes of varying calibers contained cytoskeletal elements, polysomes, distinct small ER cisterns, and elongated mitochondria with a dark matrix. In some processes, the cytoplasm was more electron-dense and contained a significant number of various organelles. In other processes, the cytoplasm was depleted, with almost no organelles and only fibrillar material of cytoskeletal elements in it. Probably, these differences were related to reorganization of the processes: formation of new processes and degeneration of old ones.

Vacuoles were sporadic and probably of mitochondrial origin, as mitochondria with clearings at various stages of transformation into vacuoles were found near them.

### Ultrastructure of astrocytes differentiated from iPSCs from a PD patient

**Figure 3.**
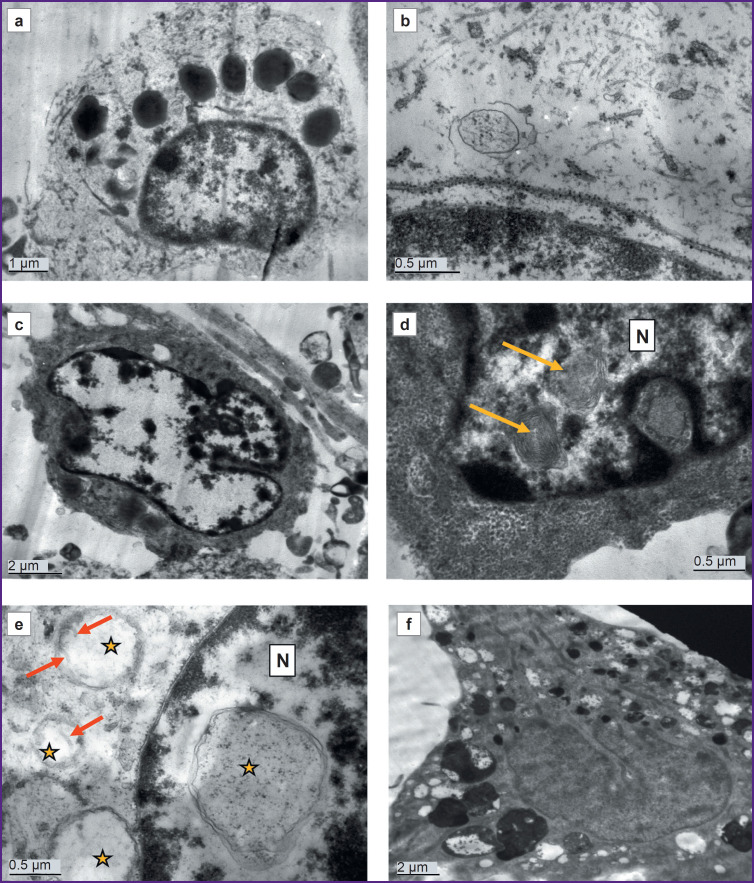
Electron microscopy of a DIV 26 astrocyte culture obtained from cell material from a donor with a mutation in the *LRRK2* gene: (a, b) cells degenerating according to the light type; electron-transparent cytoplasm, with large lipid droplets, single fragments of the cytoskeleton and organelles; no mitochondria; (c, d) cells degenerating according to the dark type; electron-dense cytoplasm, containing single degenerating ribosomes and other organelles; lipid droplets and lipophagosomes are available, no mitochondria; multilamellar structures are visible in the nucleus (N) (*yellow arrows*); (e) double-membrane vacuoles are seen both in the nucleus (N) and in the cytoplasm (*yellow stars*); the latter are hypothetically formed from mitochondria, as single cristae (*red arrows*) are visible in them; (f) a cell filled with autophagolysosomes with mixed electron-dense and electron-transparent contents

**Figure 4.**
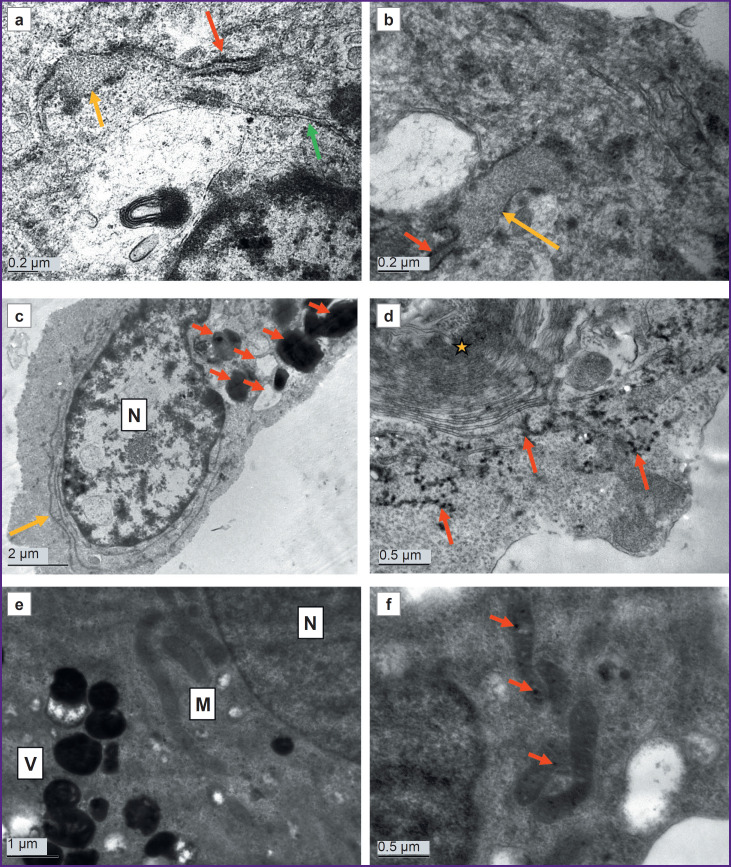
Electron microscopy of a DIV 26 astrocyte culture obtained from cell material from a donor with a mutation in the *LRRK2* gene: (a, b) changes in the granular endoplasmic reticulum; *red arrow* — a section of a cistern with normal diameter; *yellow arrow* — abnormally dilated section of the granular endoplasmic reticulum filled with moderately electron-dense contents; *green arrow* — abnormally narrowed section of the granular endoplasmic reticulum void of ribosomes; (c) long cisterns of the granular endoplasmic reticulum (*yellow arrow*) are concentrically twisted around the nucleus (N); various large vacuoles (*red arrows*) in the cytoplasm; no mitochondria; (d) concentrically twisted lamellar structures (*yellow star*), presumably formed from the endoplasmic reticulum; electron-dense inclusions of glycogen are seen nearby (*red arrows*); (e) a cell with electron-dense cytoplasm, autophagic vacuoles (V), and elongated mitochondria (M) with a dark matrix; N — nucleus; (f) elongated mitochondria with a dark matrix and calcium inclusions (*red arrows*)

**Figure 5.**
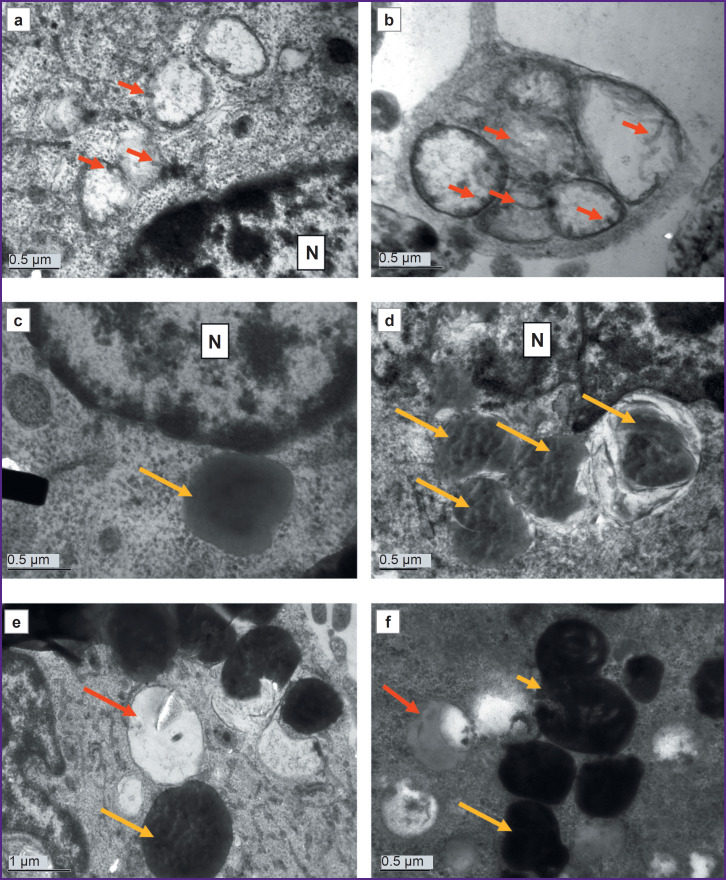
Electron microscopy of a DIV 26 culture of astrocytes obtained from cell material of a patient with a mutation in the *LRRK2* gene: (a, b) swollen mitochondria transformed into vacuoles with electron-transparent content; the mitochondrial origin of these vacuoles is suggested by the double membrane and small sporadic cristae along the periphery (*red arrows*); N — nucleus; (c) lipid droplet (*yellow arrow*) with heterogeneously moderately electron-dense content and a single membrane; N — nucleus; (d) lipophagosomes (*yellow arrows*) surrounded by at least two membranes; N — nucleus; (e) electron-transparent, fluid-filled vacuoles (*red arrow*); vacuoles with electron-dense protein-lipid content (*yellow arrow*) in a cell completely void of mitochondria; (f) various autophagolysosomes containing partially lysed lipid droplets (*red arrow*); protein content (*long yellow arrow*) and numerous compressed degenerating membranes (*short yellow arrow*)

Cultures of cells with a mutation in the *LRRK2* gene, which were cultured for the same periods as the controls (DIV 26 — PD, DIV 25 — control) demonstrated signs of significant degenerative changes (Figures 3–5). In some cells, the cytoplasm was electron-transparent and depleted, it contained sporadic elements of the cytoskeleton and short fragments of the ER cisterns, while the cytolemma was partially or completely destroyed (Figure 3 (a, b)). In other cells, on the contrary, a moderately electron-dense fine-grained material was seen in the cytoplasm, apparently demonstrating coagulating proteins of the cytosol and cytoskeleton (Figure 3 (c, d)). The nuclei of most cells contained flocculent material in the karyoplasm, clumps of condensed chromatin, and membrane inclusions that formed bubbles and multilamellar structures (see Figure 3 (c, d); Figure 3 (e)). The cisterns of the granular ER often had no ribosomes; detached ribosomes were located in the cytoplasm near the cisterns (Figure 4 (a, b)). Dilated cisterns of the granular ER filled with moderately electron-dense contents were frequently seen. Cisterns of the granular ER with abnormally narrowed internal space were also found. The granular ER often formed 1–3 continuous rings around the nucleus (Figure 4 (c)). Some cells contained concentric structures consisting of cisterns or tubules; glycogen granules were found around some of them (Figure 4 (d)).

Mitochondria were undetectable in many cells. Instead, vacuoles containing varying electron densities were available (see Figure 3 (a, c); see Figure 4 (c)). The surviving mitochondria were small, elongated, with a dark matrix and poorly distinguishable cristae and dark granules — calcium inclusions (Figure 4 (e, f)).

Vacuoles of various electron densities constituted the most characteristic element of the ultrastructure of astrocytes with the PD-associated mutation at DIV 26 (see Figure 3 (a, c, f); Figure 5). Some vacuoles appeared empty, while others had two membranes, the inner membrane sometimes forming isolated invaginations that resembled cristae, which suggested their formation from mitochondria degenerated by swelling (see Figure 3 (e); Figure 5 (a, b)). Other vacuoles had a single membrane and moderately electron-dense, unevenly stained contents and represented lipid droplets (Figure 5 (c)). The third type of vacuoles contained unevenly stained electron-dense material inside, probably of a mixed lipid-protein nature (Figure 5 (e)). Autophagolysosomes containing products of partial digestion of membrane organelles and lipid droplets were numerous (see Figure 3 (f); see Figure 4 (e); Figure 5 (d, f)).

**Figure 6.**
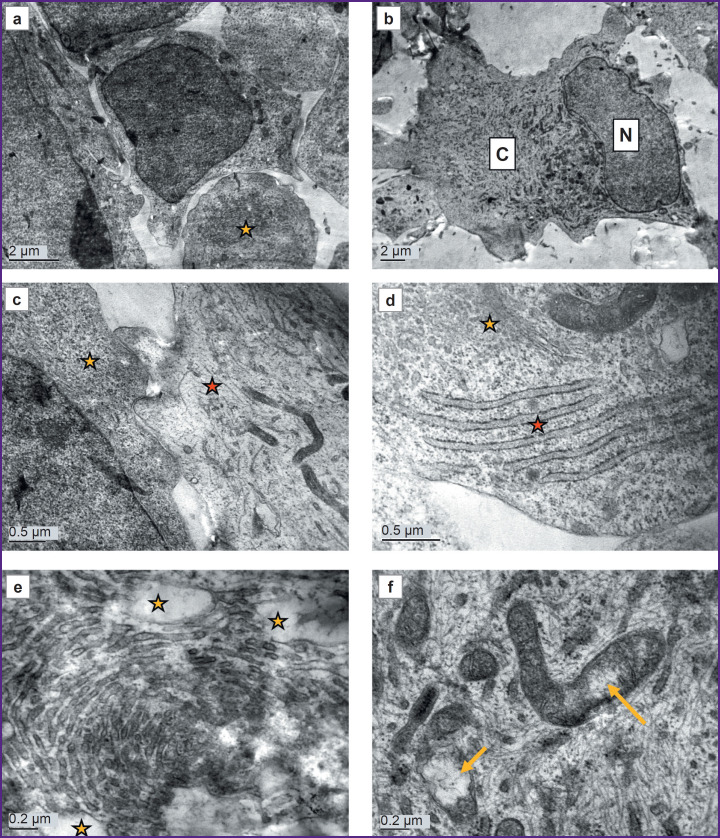
Electron microscopy of a DIV 10 astrocyte culture obtained from cell material from a donor with a mutation in the *LRRK2* gene: (a) cells of various sizes and shapes, with a relatively normal ultrastructure; one of the cells has a significant conglomerate of cisternae, probably, from smooth endoplasmic reticulum (*yellow star*); (b) a large cell with a small nuclear-cytoplasmic ratio, with voluminous cytoplasm (C) rich in organelles; N — nucleus; (c) two cell types with different ultrastructures; the left cell (*yellow star*) has darker cytoplasm, poor in cytoskeletal elements and rich in polysomes; the right cell (*red star*) has a more electron-transparent cytoplasm, well-developed cytoskeletal elements, and dark, elongated mitochondria; (d) a cell resembling a neuron in ultrastructure; a well-developed Golgi apparatus (*yellow star*); a stack of cisternae of the granular endoplasmic reticulum (*red star*), resembling a Nissl body typical for neurons; (e) concentrically located cisternae of the smooth endoplasmic reticulum with swelling areas (*yellow stars*); (f) swelling mitochondria with clearing areas (*yellow arrows*)

As the cells cultured from a PD patient at DIV 26 demonstrated ultrastructural degeneration typical of a necrobiosis state, the authors additionally examined the cells of this line at a shorter culture period (DIV 10) to assess the dynamics of such pathological changes. Ultrastructurally, the cells at DIV 10 differed significantly from the control (Figure 6). The cultures were morphologically heterogeneous. Together with large glial cells (Figure 6 (b, c), *red star*), there were both small, poorly differentiated cells (Figure 6 (c), *yellow star*) and cells with an ultrastructural similarity to neurons: they had more developed organelles, including orderly stacks of granular ER cisterns resembling Nissl bodies (Figure 6 (d)). Cells with hypertrophied cisterns of the smooth endoplasmic reticulum or Golgi apparatus were frequently seen. The cisternae were concentrically twisted and sometimes occupied a significant area of a cell (Figure 6 (e)). These cisternae swelled at some sites, giving rise to electron-transparent vacuoles. The cytoskeleton consisted of short, randomly arranged fragments of intermediate filaments and microtubules (Figure 6 (f)).

The protein-synthesizing apparatus in some parts of cells consisted of a well-developed granular ER and polysomes; in other parts, on the contrary, it was poorly developed and consisted mainly of free, inactive ribosomes.

The cells contained both normal mitochondria and swollen mitochondria, with destroyed cristae and electron-dense calcium inclusions (see Figure 6 (f)).

**Figure 7.**
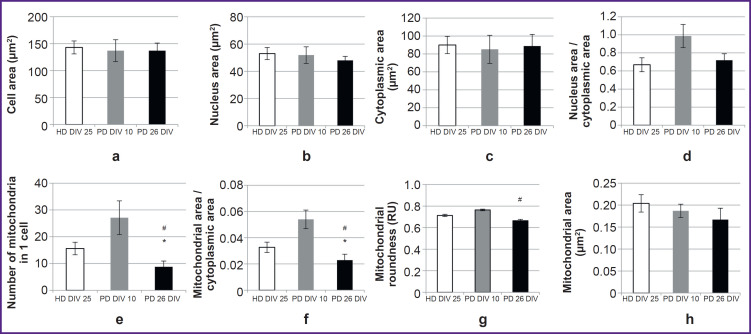
Morphometric study of astrocytes at DIV 25 (a–h) obtained from cell material from a healthy donor, and astrocytes at DIV 10 and DIV 25 obtained from cell material from a patient with a mutation in the *LRRK2* gene Data is shown as the mean ± SEM. HD DIV 25 — healthy donor, 25 days *in vitro*; PD DIV 10 — donor with PD, 10 days *in vitro*; PD DIV 26 — donor with PD, 26 days *in vitro*. Comparisons were made between the HD DIV 25 and PD DIV 26 groups and between the PD DIV 26 and PD DIV 10 groups. Differences were considered statistically significant at p≤0.05 (Mann–Whitney test); ^*^ statistically significant difference between the HD DIV 25 and PD DIV 26 groups; ^#^ between groups PD DIV 26 and PD DIV 10

The authors morphometrically assessed various cell parameters in all studied cultures and compared these parameters between cells from a PD patient at DIV 26 and cells from a healthy donor of the similar culturing period (DIV 25), as well as between cells from a PD patient at DIV 26 and cells of the same line at DIV 10 (Figure 7). The cells showed no differences in mean cell area, nuclear area, cytoplasmic area, and nuclear-cytoplasmic ratio (Figure 7 (a–d)).

Differences in the mean values of mitochondrial morphometric parameters were detected in the cells of the studied cultures. In the culture with the PD-associated mutation at DIV 26, the number of mitochondria per cell and the relative area occupied by mitochondria in the cytoplasm were significantly lower compared to the control (Figure 7 (e, f)). At that, the average value of these parameters in the astrocyte culture with the PD-associated mutation at DIV 10 was higher than in the culture of the same line at DIV 26. The histogram (see Figure 7 (e, f)) demonstrates that these values in the PD culture at DIV 10 were slightly higher compared to the control. However, the authors did not compare these two cultures due to a difference in cultivation time; any differences identified between them can rather be attributed to differences in the timing of differentiation than to pathological features of the culture with the genetic mutation. In the PD culture at DIV 25, the mitochondrial circularity value was lower than in the PD culture at DIV 10, but did not differ from the control (Figure 7 (g)). The mitochondrial area in PD culture cells at DIV 25 was slightly smaller than in the control and in PD culture at DIV 10, but these differences were not statistically significant (Figure 7 (h)).

## Discussion

This study was dedicated to astrocyte cultures obtained by directed differentiation from iPSCs. The development of a technique to reprogram somatic cells into iPSCs followed by directed differentiation into any desired cell type was a revolution for *in vitro* techniques; it is currently one of the most rapidly developing areas of biomedical research in the world. Differentiation protocols for each cell type are constantly updated and improved. The protocol developed and described in this study is based on guidelines outlined in the study of 2018 [19]; it ensures stable production of viable astrocyte cultures, the phenotype of which was confirmed by authors in this study using a combination of techniques: real-time PCR, immunocytochemical staining, and TEM. In both the control and PD-associated mutation cell lines, the cells expressed glia-specific proteins, and their ultrastructure generally corresponded to the ultrastructure of human gray matter astrocytes described in the literature [20]. However, in the culture of astrocytes with the PD-associated mutation in the *LRRK2* gene, cells ultrastructurally corresponding to developing neurons were seen. In an earlier published study [14], the authors described a more significant increase in the transcription of the β-III-tubulin gene, specific for neurons, during long-term culturing of astrocytes with the *LRRK2* mutation compared to control astrocytes. The data indicate the possibility of a certain number of cells differentiated in the neuronal direction in the culture of astrocytes obtained from iPSCs in line with the applied protocol. Moreover, a PCR study provided that some glia-specific genes were expressed at reduced levels in cells carrying the mutant gene, which may indicate a delay in or a disruption of the differentiation process in glial cells from a PD patient. The conducted morphometric study found no differences in cell size or the nuclear-cytoplasmic ratio between cells from a healthy donor and cells from a PD patient. Other morphometric data were obtained in [21]: astrocytes with the G2019S mutation in the *LRRK2* gene were significantly smaller in size compared to controls.

The results of the electron microscopy examination in the present study indicate distinct changes in the ultrastructure of astrocytes with a PD-associated mutation in the *LRRK2* gene, ultimately leading to the death of these cells in case of long culturing. Increased levels of apoptosis were demonstrated earlier in studies on neuroblastoma cultures and mouse cortical neurons with a mutation in the *LRRK2* gene [22]. However, in this study, classical ultrastructural signs of apoptosis, such as structured condensation of chromatin and organelles and formation of apoptotic bodies, were not seen in cells with the *LRRK2* mutation during long culturing (DIV 26). The cells were in a necrobiotic state with typical irreversible changes in the nucleus and organelles: destruction of chromatin, termination of protein synthesis with detachment of ribosomes from the cisterns of the granular ER and their breaking into separate subunits, disintegration of the cytoskeleton and cell membranes, and disappearance of mitochondria. The latter was the most pronounced, indicating that mitochondria were subject to the most critical changes in case of mutations in the *LRRK2* gene. Destruction of mitochondria resulted in distorted fatty acid oxidation in the cell and formation of lipid and protein inclusions, as well as caused ATP production termination and, thus, cessation of all energy-dependent intracellular processes. Astrocytes can synthesize and accumulate a small amount of glycogen [23]. In this study, the cells in cultures of astrocytes with PD (in contrast to the control) had significantly more developed smooth ER cisterns and glycogen inclusions, indicating compensatory and adaptive cellular reactions in response to a decrease in ATP production due to mitochondrial destruction. Accumulated glycogen allows astrocytes to maintain vital functions for some time through glycolysis, but glycogen reserves are not endless. A decrease in mitochondrial activity and ATP production with a simultaneous increase in the glycolytic process in astrocytes obtained from iPSCs from cellular material of patients with PD with the G2019S mutation in the *LRRK2* gene was also reported in [21].

In an earlier published study, the authors demonstrated pathological changes in mitochondria in a neuronal culture differentiated from iPSCs from the same donor with a mutation in the *LRRK2* gene [24]. Mitochondria in neurons were at different stages of cristae destruction, swelling, and transformation into electron-transparent vacuoles. In the same study, using the Nanostring multiplex gene transcriptional activity measurement technique, the authors revealed changes in the activity of genes associated with functioning of the respiratory chain, ATP production, mitochondria-ER interaction, maintenance of intracellular calcium balance, mitophagy, and mitochondrial DNA replication in neurons of PD patients [24]. Mitochondrial abnormalities were identified not only in neurons and glia, but also in fibroblasts with a mutation in the *LRRK2* gene [25]. These abnormalities appeared as a universal and leading sign of PD associated with this mutation. Several other studies also demonstrated changes in mitophagy levels in cultures with a mutation in the *LRRK2* gene. For example, in [26] authors showed a decrease in the number of mitochondria in dendrites as a result of an increase in mitophagy due to a calcium imbalance. In this study, a decrease in the number of mitochondria up to a complete disappearance thereof in culture at DIV 26 happened not mainly through mitophagy, but as a result of the gradual destruction of the mitochondria themselves, their swelling, and transformation into vacuoles. A possible cause of mitochondrial dysfunction in astrocytes with the G2019S mutation in the *LRRK2* gene may be endoplasmic reticulum stress [27], indicating a disruption and termination of post-translational changes and further intracellular transport of proteins synthesized in the granular ER. The authors identified the following ultrastructural signs of endoplasmic reticulum stress in the study: the appearance of dilated cisterns filled with moderately electron-dense contents and the detachment of ribosomes from the granular ER. Here, enhanced contact formation between the dysfunctional ER and mitochondria in this condition leads to mitochondrial overload with Ca^2+^ ions, their damage [27], and subsequent initiation of cell death [28].

Another typical pathology observed in glial cells from a PD patient in this study was the emergence of vacuoles with varying electron density contents. Such vacuoles were most often seen in cells completely depleted of mitochondria during long culturing (DIV 26). The authors believe that vacuole development is directly related to mitochondrial degeneration.

Large vacuoles with electron-transparent contents, possibly, formed as a result of mitochondrial swelling. Higgins et al. [29] described cytoplasmic vacuolization in mice model of amyotrophic lateral sclerosis. By means of immunofluorescence and immunoelectron microscopy, the authors demonstrated that vacuoles formed through mitochondrial swelling rather than through autophagy. The process of swelling, destruction of cristae, and gradual transformation of mitochondria into vacuoles was also described in such cardiomyocyte pathology as mitochondrial vacuolar degeneration [30].

The accumulation of lipid droplets in cells is considered a sign of stress caused by a variety of external and internal factors [31]. Lipid droplets were also found in neurons with the same mutation in the *LRRK2* gene [32]. An increase in the number of lipid droplets in astrocytes during mitochondrial dysfunction, hypoxia, and formation of reactive oxygen species is an adaptive, protective, and evolutionarily conserved mechanism being an early sign of the neurodegenerative process [33, 34]. The lipid droplets and lipophagosomes identified in this study in dying cells could develop either as a result of an increase in the level of their synthesis in the endoplasmic reticulum or as a result of decomposition (phanerosis) of degenerating mitochondria and other membrane organelles; however, these ideas must be confirmed using molecular biology techniques. In recent years, a number of studies demonstrated the localization of the *LRRK2* gene expression product, dardarin, in close connection with membrane-bound organelles and cellular vesicles and its participation in lipid metabolism, including the phosphorylation and catabolism of plasma membranes and lysosomal lipids [35]. Thus, a mutation in the *LRRK2* gene may be related to a dysfunction of the normal utilization of cellular organelle membranes and accumulation of degradation products in the form of lipid and protein inclusions.

The accumulation of autophagolysosomes demonstrated in this study indicates a dysfunctional macroautophagy in cells with a mutation in the *LRRK2* gene and is consistent with numerous data in literature [7–11, 17].

To assess the dynamics of pathological changes in astrocytes with a mutation in the *LRRK2* gene, the authors compared the ultrastructure of long-cultured (DIV 26) cells with cells of the same lineage cultured for a shorter period (DIV 10). Morphometric parameters (profile field area, nuclear area, and nuclear-cytoplasmic ratio) of these cultures were similar. In the culture at DIV 10, the ultrastructure of the cells was more preserved, with only a few cells demonstrating signs of upcoming cell death. Moreover, cells in this culture demonstrated hyperplastic compensatory and adaptive changes, such as hypertrophy of the synthetic apparatus and an increase in the number of mitochondria. Probably, by day 26 *in vitro*, the adaptive reserve was exhausted, and the culture cells at DIV 26 showed only ultrastructural signs of degeneration.

The morphometric study demonstrated that in cultures with the PD-associated mutation (DIV 26), the average number of mitochondria per cell was statistically significantly lower than in the control. These differences were to a great extent explained by the fact that many cells in the culture with the PD-associated mutation had no mitochondria: they were changed beyond recognition. However, in terms of morphometric parameters (size and elongation), the remaining mitochondria did not differ much from the control. In both groups, mitochondria were predominantly small and oval. In cells with the *LRRK2* mutation in culture DIV 10, the number and area of mitochondria were higher than in cells in culture DIV 26, which is a sign of compensatory and adaptive reactions over a shorter period of cultivation.

The results of this electron microscopic study demonstrate a significant reduction in mitochondrial viability in cells with a mutation in the *LRRK2* gene, which is consistent with the literature data [24–26] on the critical role of mitochondria in the PD pathogenesis. The accumulation of various vacuoles is a typical phenotypic feature of cells with this mutation; it indicates impaired autophagy. Further research is required to clarify the molecular mechanisms leading to the observed ultrastructural changes in cells with a mutant *LRRK2* gene.

## Conclusion

In this study, the phenotype of astrocytes differentiated from iPSCs was confirmed using a combination of real-time PCR, immunocytochemical staining for glia-specific proteins, and TEM. Both PCR and TEM identified abnormalities in the differentiation process in the cell line with the PD-associated mutation. Here, electron microscopy allowed finding ultrastructural abnormalities in the mutant cell line that were not visible during routine phase-contrast microscopy of living cells and fluorescence microscopy of immunofluorescence-stained cells. The abnormalities identified by TEM include mitochondrial damage, impaired autophagy, and endoplasmic reticulum stress. These pathologies can also be identified using molecular genetic techniques, including PCR, Nanostring, and etc. However, electron microscopy is significantly less expensive and does not require various reagents for different tasks, as the case is with specialized Nanostring panels or PCR primers. TEM examination allows for simultaneous observation of the morphofunctional state of all cell components (the nucleus, cytoplasm, and organelles) in a single section. Therefore, electron microscopy can also be used as a primary examination technique at the initial analysis of a pathological sample to identify vectors for further molecular, genetic, and functional examination using other techniques.

Thus, TEM demonstrates a significant potential for use in studies related to cellular technologies, both as a standalone technique and in combination with traditional techniques including PCR, immunocytochemical staining for glia-specific proteins, and etc.
